# Mapping of leptin and its syntenic genes to chicken chromosome 1p

**DOI:** 10.1186/s12863-017-0543-1

**Published:** 2017-08-09

**Authors:** Eyal Seroussi, Frédérique Pitel, Sophie Leroux, Mireille Morisson, Susanne Bornelöv, Shoval Miyara, Sara Yosefi, Larry A. Cogburn, David W. Burt, Leif Anderson, Miriam Friedman-Einat

**Affiliations:** 1 Department of Animal Science, Agricultural Research Organization, Volcani Center, P.O. Box 15159, 7528809 Rishon LeTsiyon, Israel; 2 GenPhySE, Université de Toulouse, INRA, INPT, ENVT, 31326 Castanet Tolosan, France; 3 Department of Medical Biochemistry and Microbiology, Uppsala University, SE-75123 Uppsala, Sweden; 4 Department of Animal and Food Sciences, University of Delaware, Newark, DE 19716 USA; 5 The Roslin Institute and Royal (Dick) School of Veterinary Studies, University of Edinburgh, Midlothian, EH25 9RG UK; 6 Department of Veterinary Integrative Biosciences, College of Veterinary Medicine and Biomedical Sciences, Texas A&M University, College Station, TX 77843-4458 USA; 7 Department of Animal Breeding and Genetics, Swedish University of Agricultural Sciences, SE-75007 Uppsala, Sweden

**Keywords:** Chicken leptin, Syntenic clusters, chicken RBM28, GC-rich, chicken chromosome 1

## Abstract

**Background:**

Misidentification of the chicken *leptin* gene has hampered research of leptin signaling in this species for almost two decades. Recently, the genuine leptin gene with a GC-rich (~70%) repetitive-sequence content was identified in the chicken genome but without indicating its genomic position. This suggests that such GC-rich sequences are difficult to sequence and therefore substantial regions are missing from the current chicken genome assembly.

**Results:**

A radiation hybrid panel of chicken-hamster Wg3hCl2 cells was used to map the genome location of the chicken *leptin* gene. Contrary to our expectations, based on comparative genome mapping and sequence characteristics, the chicken *leptin* was not located on a microchromosome, which are known to contain GC-rich and repetitive regions, but at the distal tip of the largest chromosome (1p). Following conserved synteny with other vertebrates, we also mapped five additional genes to this genomic region (*ARF5*, *SND1*, *LRRC4*, *RBM28*, and *FLNC*), bridging the genomic gap in the current Galgal5 build for this chromosome region. All of the short scaffolds containing these genes were found to consist of GC-rich (54 to 65%) sequences comparing to the average GC-content of 40% on chromosome 1. In this syntenic group, the RNA-binding protein 28 (*RBM28*) was in closest proximity to *leptin*. We deduced the full-length of the *RBM28* cDNA sequence and profiled its expression patterns detecting a negative correlation (*R* = − 0.7) between the expression of *leptin* and of *RBM28* across tissues that expressed at least one of the genes above the average level. This observation suggested a local regulatory interaction between these genes. In adipose tissues, we observed a significant increase in *RBM28* mRNA expression in breeds with lean phenotypes.

**Conclusion:**

Mapping chicken *leptin* together with a cluster of five syntenic genes provided the final proof for its identification as the true chicken ortholog. The high GC-content observed for the chicken *leptin* syntenic group suggests that other similar clusters of genes in GC-rich genomic regions are missing from the current genome assembly (Galgal5), which should be resolved in future assemblies of the chicken genome.

**Electronic supplementary material:**

The online version of this article (doi:10.1186/s12863-017-0543-1) contains supplementary material, which is available to authorized users.

## Background

The chicken *leptin* gene was recently identified, more than 20 years after the first identification of mammalian *leptin* [1]. An extensive search for chicken leptin [2–5] led to erroneous identification, characterization and mapping ([4, 6–10]. While the erroneous mapping was retracted [11], the erroneous sequences of chicken, turkey and duck *leptins* were not withdrawn [GenBank: AF012727, AAC32381 and AAT38807], respectively. The high GC content of avian *leptins* [74 ± 2 (SE) %] is significantly higher than that in *leptins* from non-avian vertebrates [52 ± 3 (SE) %] [1] and contains repetitive and palindromic sequence elements. This observation pointed to the possibility that the avian *leptins* may be located on microchromosomes, which are known to contain these types of sequence elements and, in addition, are underrepresented in avian genomes assemblies [12, 13]. For example, the average GC-content in chicken chromosome 1 is 40.3% compared with an average of 56.5% for chromosomes 25 to 33 [GenBank: GCA000002315.3]. High-quality genome assemblies for chicken and other birds revealed a large proportion of missing genes compared to other vertebrates [14, 15]. The presumed loss of the protein-coding genes was estimated to be as high as 30% [14]. These missing genes often form synteny groups, which are conserved among vertebrates. Therefore, it has been suggested that these genes were lost from avian genomes during the evolutionary process when microchromosomes were formed in the avian lineage [14, 15]. However, technical difficulties in identification of some avian genes, due to their sequence characteristics and high sequence divergence, have been suggested as an alternative explanation [1, 16–18]. Recently, the submission of a new version of the chicken genome assembly (Galgal5) revealed 1811 new protein-coding genes, however a fairly large number of genes belonging to clusters of genes showing conserved synteny in other species are still missing in the latest assembly [19]. To better understand this phenomenon, it is important to characterize novel genes that were considered missing, especially those like *leptin* and its syntenic genes, most of which are missing in Galgal5. The mapping of *leptin* and characterization of its synteny group are essential; both as a final proof for the identification of chicken *leptin* and for shedding light on possible technical barriers that hamper identification of genes in the chicken genome and in other genome assemblies.

The chicken genome assembly reveals some conserved synteny between human chromosome 7 and chicken chromosome 1, including genes flanking *leptin* and a cluster of closely linked genes on both sides on human chromosome 7 [4]. However, other gene orthologs within this region on chicken chromosome 1 map to human chromosome 22. This break in synteny gave ground to the hypothesis that *leptin*, together with flanking genes, could have been translocated onto another chicken chromosome, possibly a microchromosome. Here, we demonstrate using radiation hybrid (RH) mapping that *leptin* and six other closely-linked genes are in fact located in a highly GC-rich region on the distal tip of chicken chromosome 1p.

## Results

### Mapping the chicken *leptin* gene and six other closely-linked genes

**Fig. 1:**
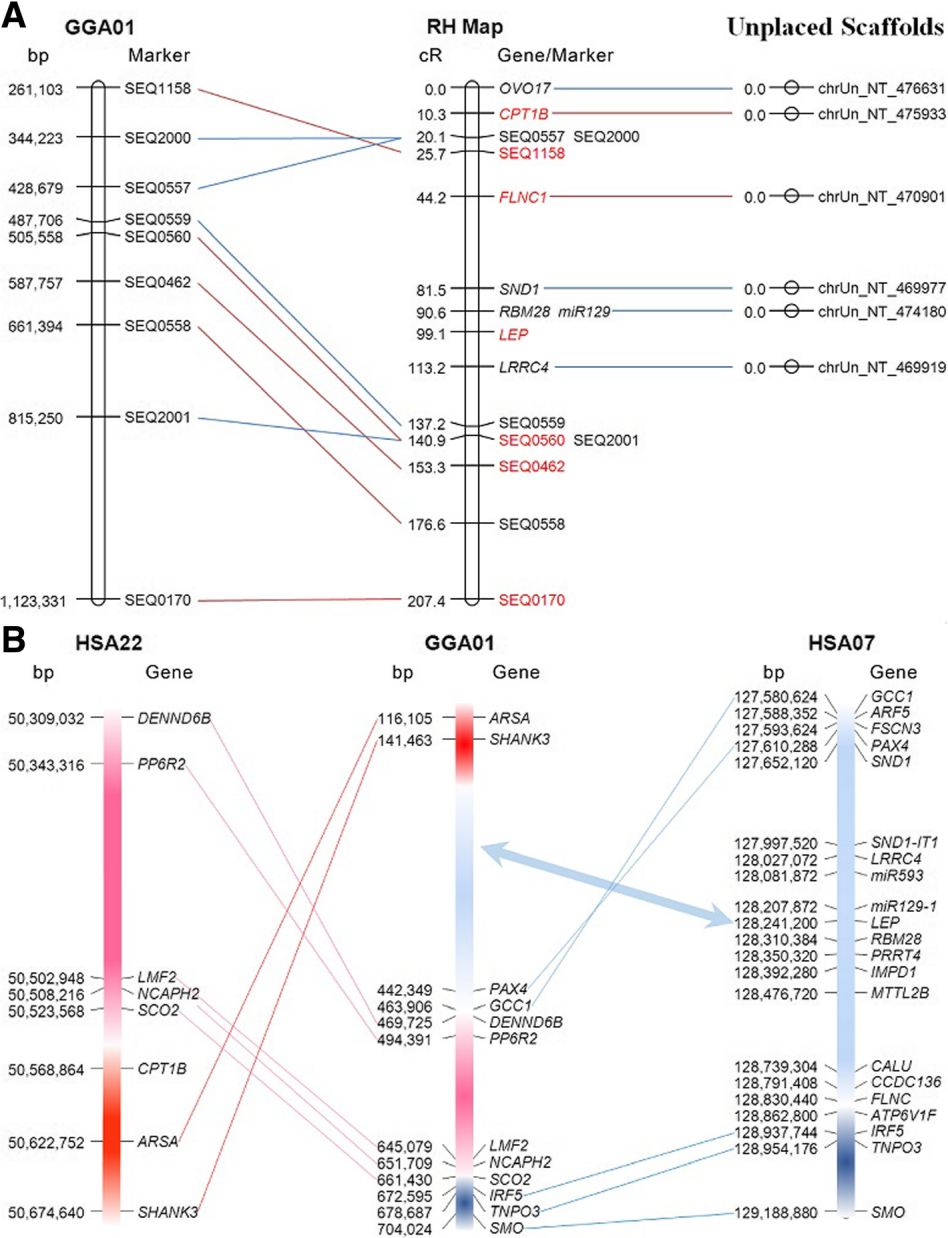
Mapping of leptin and its syntenic genes to chicken chromosome 1p. **a** Comparison between the RH map and the relevant genomic regions in chicken (Galgal5) and human genome assemblies. The RH map obtained in this study (RH Map) is compared to the chicken chromosome 1 (*GGA*01) assembly in Galgal5. Markers localized on unplaced scaffolds are indicated on the right. *Red and blue colors* indicate marker mappings with relatively higher or lower likelihoods, respectively (LOD scores are indicated in Additional file 1: Table S1). **b** Synteny conservation between the distal part of chicken chromosome 1p (GGA01) and human chromosomes 22 (HSA22, *reddish colors*) and 7 (HSA07, *blueish colors*)

We mapped *leptin* using the well-established chicken RH panel in Wg3hCl2 cells, prepared by fusing chicken embryonic diploid fibroblasts with hypoxanthine-guanine phosphoribosyl transferase (HPRT)-deficient hamster cells [20, 21]. This analysis demonstrated that chicken *leptin* is located between markers SEQ0557 (428,679 bp) and SEQ559 (487,706 bp) at the distal tip of chromosome 1p (Fig. 1; Additional file 1: Table S1).

To corroborate this mapped position of *leptin*, we explored its co-localization with the orthologs of human genes located in the near vicinity of *leptin*. Using a BLASTN search, we identified poorly annotated chicken orthologs of *RBM28*, *SND1*, *LRRC4*, and *FLNC* in short unplaced scaffolds in Galgal5, which we annotated in a parallel study [16]. *miR-129a* was found by blast in NT474180, and *ARF5* was annotated in a short unplaced scaffolds in Galgal5. These leptin syntenic genes and their genomic scaffolds were found to have high GC-content of ~60% (Table 1). This signifies that these sequences have a lower complexity and a higher incidence of repetitive and palindromic sequences, all of which contribute to the technical difficulty of identification and mapping of GC-rich sequences in Galgal5.

**Table 1:** Unplaced GC-rich genomic scaffolds containing genes from the *leptin* synteny group

Galgal5 Scaffold	% GC	Length (bp)	Gene
NT475614	54	3840	*ARF5*
NT474055	65	5035	*SND1*
NT470859	62	8296	*LRRC4*
NT470901	58	8252	*FLNC*
NT474180	62	4926	*miR129a*

Based on the sequence information gathered for these genes, PCR primers were designed (Additional file 1: Table S1) and the resulting amplicons were RH mapped. The analysis confirmed the close localization of these genes and leptin, which is consistent with co-localization of the human orthologs on human chromosome 7 (Fig. 1a-b). *CPT1B*, previously mapped to chicken chromosome 1 [22], was used as an additional positive control for the RH mapping and the synteny break on HSA22 (Fig. 1b).

### Characterization of *RBM28* structure and expression

**Fig. 2:**
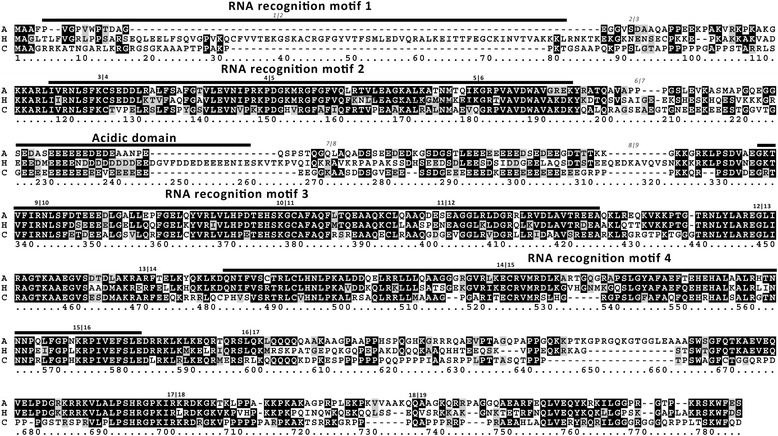
Comparison of the predicted amino-acid sequence deduced for chicken *RBM28* to those of alligator and human. The amino-acid sequence of alligator (A, *Alligator mississippiensis*, [GenBank: XM014610288] was aligned to human (H, *Homo sapiens,* [GenBank: KR710309]), and chicken (C; PRJEB18741). Dashes indicate gaps in the alignment. Identical, similar, and non-conserved residues are indicated by a *black, grey,* and *white background*, respectively. Exon borders of the human gene are indicated by the exon numbers in bold (conserved between species) or italics (non-conserved), based on the exon comparison table (Additional file 2: Table S2). RNA recognition motifs 1–4 and the acidic domain, characterized for the human *RBM28* [26] are delineated above the sequence

In vertebrate genomes for which leptin and its syntenic group of genes are annotated, *RBM28* is mapped adjacent to *leptin*. In most cases, it is in a tail-to-tail orientation (3′ to 3′; e.g. peregrine falcon, *Falco peregrinus* [23, 24]; fugu, *Takifugu rubripes* [GenBank Gene ID: 548,631]; green sea turtle, *Chelonia mydas* [GenBank Gene ID: 102,932,266]; and mouse, *Mus musculus* [GenBank Gene ID: 16,846]). Moreover, we have previously shown that *leptin* is very closely linked to *RBM28* in peregrine falcon and fugu (1.8 and 0.4 kbp, respectively [23]). Since such close proximity and the conserved 3′ to 3′ orientation suggest a local regulation of transcription [25], we further characterized *RBM28* and its expression pattern. We deduced the full-length cDNA of *RBM28*, using genomic and RNA-seq data available in GenBank (Fig. 2; Additional file 2: Fig. S1 and Table S2).

The assembled 2007 bp cDNA [ENA Project ID: PRJEB18741] was partially corroborated by a match to a single expressed sequence tag (EST, 838 bp, [GenBank: DR425791]). With 69% GC-content, this putative transcript was found to be capable of encoding a 668 aa polypeptide with 50–51% identity and 65% similarity to human and alligator *RBM28* [GenBank: NP001159607; GenBank: XP006033669] (Fig. 2). Higher amino acid identity of close to 90% was observed within the 3 RNA Binding Motifs (RBMs), which aligned with RBMs 2–4 of the human ortholog, each of about 90 amino acids long. Chicken and alligator proteins had a structure layout consisting of only 3 RBMs suggesting that such layout is common to reptiles, turtles (e.g. green turtle [GenBank Gene ID: 102,932,266]) and to birds (e.g. peregrine falcon [GenBank Gene ID: 102,049,081], while in mammals it retained the 4 RBM composition that is present in fish (e.g. fugu, [GenBank Gene ID: 548,631]). The acidic region between RNA recognition motifs 2 and 3, reported for the human *RBM28* [26], was found to be only partially conserved in the deduced proteins of chicken and alligator, but it was followed by an additional acidic region in chicken *RBM28* (annotated 281–309, Fig. 2). Among the 18 exon-intron junctions, 13 were found to be conserved between the chicken and the human genes (Fig. 2 and Additional file 2: Table S2).

**Fig. 3:**
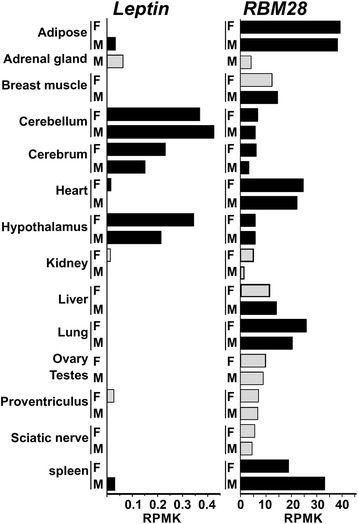
Bioinformatic analysis of *RBM28* and *leptin* expression patterns. On the right, *RBM28* cDNA was used as bait to screen RNA-seq data from adult male (M) and female (F) *red junglefowl* [GenBank: ERA252218]. On the left, chicken *leptin* cDNA [GeneBank: LN794245] was used as the bait sequence. Expression levels were calculated as reads per kilobase per million mapped reads (RPKM). Tissues with above average expression level of at least one of the two genes, *RBM28* and *leptin* (*black boxes*) were included in the correlation analysis (*R* = − 0.7)

We used a comprehensive RNA-seq experiment of a female and a male red junglefowl available in GenBank (Chickspress, [GenBank: PRJEB4677]) to characterize the *RBM28* expression pattern. The broad expression pattern obtained by searching the data for tissues included in this experiment (Fig. 3a) seemed compatible with the housekeeping role of *RBM28* as a nuclear component of the spliceosomal ribonucleoprotein complex [26]. Nevertheless, the variation in expression level between tissues suggests tissue-specific functional roles.

To explore the possibility that the conserved proximity of *leptin* and *RBM28* relates to their transcriptional control, the expression profile of chicken *leptin* was explored using the same RNA-seq dataset. Comparison of the two patterns revealed that the tissues with the highest expression of *RBM28* (adipose, lung, heart and spleen) had no significant *leptin* mRNA, whereas, tissues expressing *leptin* (cerebellum, cerebrum and hypothalamus) had only a basal level of *RBM28* expression (Fig. 3a-b). Thus, a significant negative correlation (*R* = −0.7) was observed between *leptin* and *RBM28* expression profiles, taking into account only tissues expressing an above average level of either *RBM28* or *leptin* (RPKM >13.1 and 0.07, respectively). In humans, *leptin* and *RBM28* are also in tail-to-tail orientation but at a distance of more than 40 kb and with no indication of reciprocal expression in the human RNA-Seq Atlas [27].

**Fig. 4:**
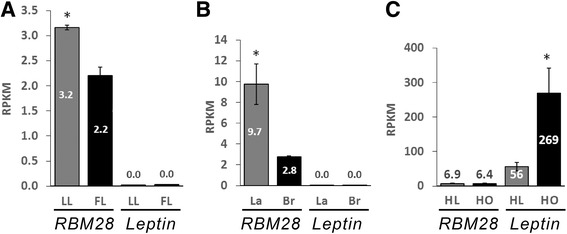
Expression of *RBM28* and *leptin* in adipose tissues of different chicken breeds and human. **a** Bioinformatic search of RNA–seq data of adipose tissue from lean (LL) and fat (FL) lines of broiler breeds at 7 weeks of age (*n* = 4 for each group; [28] GenBank: SRA062979). **b** RNA-seq analysis in 4-month old layer (La) and broiler breeder (Br) hens (*n* = 3 for each group) [16]. **c** Bioinformatic search of RNA-seq data from visceral fat of lean and obese humans (HL [GenBank: SRX470439–41], and HO [GenBank: SRX470443–45] (*n* = 3 for each group), respectively, using as bait sequence the full length cDNAs of the human *RBM28* [GenBank: NM018077] and *leptin* [GenBank: BC069452]. Results were calculated as RPKM (indicated in or above each column). Horizontal lines represent ±SEM. Asterisks indicate statistical significance (*P* ≤ 0.05)

An interesting feature was the relatively high expression level of the chicken *RBM28* in adipose tissue. As a first step to relate this expression to adipose tissue regulation, we explored the expression of *RBM28* in a previously published RNA-seq study of abdominal fat in genetically fat and lean broiler-type chickens, which exhibit a 2.8-fold difference in visceral fat deposition at 7 weeks of age [28]. The RNA-seq analysis of four lean and four fat individuals revealed a significantly higher level of *RBM28* expression in the lean line (*P* = 0.03; Fig. 4a). *Leptin* was not significantly expressed in adipose tissues of the fat and lean chickens (~0.002 RPKM). Since layer chickens, bred for efficient egg production, are substantially different from broiler type chickens in various parameters related to control of energy balance, we compared the expression of *RBM28* and *leptin* also between RNA-seq data from commercial strains of broiler (meat-type) and layer (egg-type) chickens [16]. Significantly higher levels of *RBM28* expression were observed in the commercial layer line (*P* = 0.02; Fig. 4b). Also in these two strains of chickens, leptin was not significantly expressed in visceral fat. For comparison, we searched RNA-seq dataset from obese and lean human [GenBank: SRX470443–45] and, as expected, *leptin* expression was higher in adipose tissue of obese individuals (*P* = 0.04; [29]). However, the expression of *RBM28* was not significantly different between obese and lean humans (Fig. 4c).

## Discussion

We have mapped the chicken *leptin* gene together with five closely-linked genes to chicken chromosome 1p, and demonstrated that these genes form a syntenic block in both chicken chromosome 1 and human chromosome 7. The order of the distal scaffolds on chicken chromosome 1 in Galgal5 is not in a complete accordance with the order of markers obtained from our RH mapping. Future long fragment sequencing, will hopefully resolve the correct order of genes in this region.

We found that in addition to the high GC-content of the *leptin* coding sequence (68%), the neighboring genes and genomic sequences also had high GC-content (~70% and ~60%, respectively). It is likely that the high GC-content of this cluster of genes in chickens hampered previous identification of this syntenic group [4, 11] and could explain why the *leptin* locus is missing in chicken BAC libraries (Richard Crooijmans, Wageningen University, personal communication). This finding is compatible with the known higher GC content in macrochromosomes near telomeres [30].

The chromosomal region of the chicken *leptin* synteny group and *CPT1B,* which was the gene used as a positive control for mapping and placed telomeric to the *leptin* synteny group (Fig. 1b) are closely linked to quantitative trait loci (QTLs) for performance and carcass traits [31–34]. Therefore, it is possible that *leptin*, *RBM28* and *CPT1B* are positional candidate genes for QTLs related to growth and metabolic regulation. Therefore, reassessment of mapping of these QTLs using our improved map of this region will be important to uncover a possible effect of these candidate genes on production traits.

As a representative neighboring gene of *leptin*, we chose *RBM28* for further characterization. *RBM28* is a nucleolar protein associated with small nuclear ribonucleoprotein (snRNP) and ribosome biogenesis [26]. Sequence similarity, conserved exon-intron boundaries (13 out of 18), and similarity in the domain structure with the mammalian and reptilian *RBM28* proteins, confirmed the identification of chicken *RBM28* and suggested at least partial functional conservation.

The pattern of expression obtained using red junglefowl RNA-seq data revealed its expression in all of the examined tissues, compatible with its apparent fundamental role. Nevertheless, the expression pattern also shows variability in level of expression, suggesting a tissue-specific role. Indeed, human *RBM28* deficiency is not lethal and shows some specific phenotypes including alopecia, mental retardation, progressive motor decline, and hypopituitarism (ANE syndrome) [35, 36].

The high expression of *RBM28* found in the visceral fat of chickens is intriguing due to the key role of its neighboring gene *leptin,* in the control mechanism of energy homeostasis, in mammals [1, 37]. This observation and apparent correlation of *RBM28* expression with leanness and slow body growth, suggest evolutionary adaptations in adipose tissue of birds that recruited other signaling pathways than *leptin*. The unique challenges of some avian species imposed by migration and strong seasonal variations of feeding, may have required different adaptation of the control mechanism of feed intake and fat deposition than that in mammals.

Inverse correlation between *leptin* and *RBM28* patterns of expression in red junglefowl tissue suggest opposite regulatory function such as that reported in mammalian hair follicle in mammals, where RBM28 stimulates and leptin inhibits anagen-phase development [36, 37]. This observation could help future studies of the interactions between *leptin* and *RBM28.* It is possible that their proximate chromosomal location contributes to their opposing expression pattern. In this respect, it is interesting to note that in the falcon, we have previously demonstrated that leptin and *RBM28* are less than 2 kbp apart in a tail-to-tail orientation [23]. An additional control mechanism could be through the activation of *miR-203* by *RBM28* [36], to which the leptin receptor is a potential target [38].

## Conclusions

Mapping and characterization of genes in leptin’s synteny group suggested that its depletion from genome assemblies has been due to high GC-content. Detailed analysis of the gene adjacent to *leptin*, *RBM28* and its mRNA expression pattern indicated increased transcription in adipose tissue of chicken breeds with lean phenotypes. Our observation of negative correlation between the expression patterns of chicken *leptin* and *RBM28* could be related to their adjacent chromosomal positions.

## Methods

### RH mapping

PCR amplifications were carried out for each marker (Additional file 1: Table S1) in 15 μl reactions containing 25 ng DNA from the RH panel [21], 0.4 μM of each primer, 0.25 units Taq polymerase (GoTaq, Promega), 1.5 MgCl2, 0.2 mM dNTP, using the Applied Biosystems 2720 thermal cycler. The first 5 min denaturation step was followed by 35 cycles, of denaturation at 94 °C for 30 s, annealing at Tm for 30 s and elongation at 72 °C for 30 s. Each marker was genotyped twice and a third genotyping was performed in case of discrepancy between the first two determinations. The RH map was built as previously described [39] using the Carthagene software [40] and drawn with MapChart 2.0 [41].

### Animals and tissue sampling

Female broiler (Cobb) and White Leghorn (Lohman) chickens were purchased from commercial husbandries (Brown & Sons and Hasolelim, Israel, respectively) at the age of 1 day and grown according to recommended husbandry and feeding conditions (NRC 1994) with free access to food and water. At sexual maturation, as indicated by egg lay (about 4 month of age), samples of abdominal (visceral) fat were snap-frozen in liquid nitrogen after neck dislocation. Total RNA was prepared using a RNA isolation kit (miRNeasy, Qiagen).

### RNA sequencing

cDNA libraries were prepared by the Uppsala sequencing Platform from 1 μg RNA using the TruSeq Stranded mRNA Sample Preparation Kit (Illumina, San Diego, CA) as described earlier [16]. Briefly, libraries were prepared, from visceral adipose tissue of three individual birds of each chicken breed: broiler breeder and layer hens. The libraries were uniquely tagged and paired-end sequenced (2 × 124 bp) with Illumina HiSeq sequencers, producing about 33 million paired-end reads per library.

### Bioinformatic analysis

Sequence homology searches were carried out at NCBI [non-redundant nucleotide collection (NR), SRA, and WGS] using the BLAST family of programs. Relevant sequence entries were downloaded with their quality information (FASTQ format) and reassembled using either *MIRA4* [42] or *GAP5* software [43]. Sequences were aligned using *CLUSTALW* or *MAFFT* (http://www.genome.jp/tools/clustalw) with the default parameters and the *GONNET* matrix or *E-INS-I* strategy for amino-acids and nucleotides, respectively; and colored using the *BOXSHADE* program (http://www.ch.embnet.org/software/BOX_form.html).

## Additional files

Additional file 1: Table S1. Details about the PCR primers and amplicons used for the RH mapping, sequence statistic and position. (XLSM 14 kb)

Additional file 2: Characterization of RBM28. **Figure S1.** cDNA and predicted protein sequences of RBM28. **Table S2.** RBM28 Exons in chicken alligator and human. (XLSX 13 kb)

## Abbreviations

EST: Single expressed sequence tag
Galgal5: Genome assembly *Gallus gallus* 5.0
HPRT: Hypoxanthine-guanine phosphoribosyl transferase
RBM: Binding Motifs
*RBM28*: RNA-binding protein 28
RPKM: Reads per kilobase per million mapped reads
