# Correction to: Mapping of leptin and its syntenic genes to chicken chromosome 1p

**DOI:** 10.1186/s12863-017-0587-2

**Published:** 2017-12-15

**Authors:** Eyal Seroussi, Frédérique Pitel, Sophie Leroux, Mireille Morisson, Susanne Bornelöv, Shoval Miyara, Sara Yosefi, Larry A. Cogburn, David W. Burt, Leif Andersson, Miriam Friedman-Einat

**Affiliations:** 1 Department of Animal Science, Agricultural Research Organization, Volcani Center, P.O. Box 15159, 7528809 Rishon LeTsiyon, Israel; 2 GenPhySE, Université de Toulouse, INRA, INPT, ENVT, 31326 Castanet Tolosan, France; 3 Department of Medical Biochemistry and Microbiology, Uppsala University, SE-75123 Uppsala, Sweden; 4 Department of Animal and Food Sciences, University of Delaware, Newark, DE 19716 USA; 5 The Roslin Institute and Royal (Dick) School of Veterinary Studies, University of Edinburgh, Midlothian, EH25 9RG UK; 6 Department of Veterinary Integrative Biosciences, College of Veterinary Medicine and Biomedical Sciences, Texas A&M University, College Station, TX 77843-4458 USA; 7 Department of Animal Breeding and Genetics, Swedish University of Agricultural Sciences, SE-75007 Uppsala, Sweden

## Correction

After the publication of this work [1] an error was noticed in one of the author surnames. The author name Leif Anderson should be spelt as Leif Andersson.
